# Macintosh-style videolaryngoscope use for tracheal intubation in elective surgical patients revisited: a sub-analysis of the 2022 Cochrane review data

**DOI:** 10.1186/s13037-024-00402-2

**Published:** 2024-05-28

**Authors:** Alistair F. McNarry, Patrick Ward, Ubong Silas, Rhodri Saunders, Sita J. Saunders

**Affiliations:** 1 Department of Anaesthesia, St John’s Hospital, NHS Lothian, Edinburgh, UK; 2 Coreva Scientific, Königswinter, Germany

## Abstract

The Cochrane systematic review and meta-analysis published in 2022 that compared videolaryngoscopy (VL) with direct laryngoscopy (DL) for facilitating tracheal intubation in adults found that all three types of VL device (Macintosh-style, hyper-angulated and channeled) reduced the risk of failed intubation and increased the likelihood of first-pass success. We report the findings of a subgroup re-analysis of the 2022 Cochrane meta-analysis data focusing on the Macintosh-style VL group. This was undertaken to establish whether sufficient evidence exists to guide airway managers in making purchasing decisions for their local institutions based upon individual device-specific performance. This re-analysis confirmed the superiority of Macintosh-style VL over Macintosh DL in elective surgical patients, with similar efficacy demonstrated between the Macintosh-style VL devices examined. Thus, when selecting which VL device(s) to purchase for their hospital, airway managers decisions are likely to remain focused upon issues such as financial costs, portability, cleaning schedules and previous device experience.

The role of videolaryngoscopes in facilitating tracheal intubation has been explored extensively since the first description of the GlideScope [1]. A variety of different types of videolaryngoscope now exist, which may be broadly categorized as Macintosh-style, hyper-angulated, and channeled [2]. A systematic review of 21 meta-analyses comparing videolaryngoscopy (VL) with direct laryngoscopy (DL) found the majority of meta-analyses examined had grouped together Macintosh-style and hyper-angulated videolaryngoscope blades, which the authors concluded could potentially mislead airway managers in their choice of which videolaryngoscope to employ for a particular patient or scenario [3]. In their editorial on the subject of VL versus DL, Hansel and El-Boghdadly stated that the threshold of evidence required to support the superiority of VL over DL in achieving firs﻿t-pass success (FPS) had been reached in 2015 [4], before the first Cochrane review on the topic had been published [5] ― though Hansel and El-Boghdadly did not recommend any particular VL device. Carvalho et al.*’s *subsequent network meta-analysis did attempt to rank videolaryngoscopes; however, they were unable to report any single videolaryngoscope that performed significantly better than others for preventing failed intubation [6]. The most recent Cochrane review, in 2022, considered 222 randomized controlled trials, involving 26,149 participants [7, 8]. It described four ‘critical outcomes’: failed intubation, FPS at tracheal intubation, esophageal intubation, and hypoxemia. The authors reported that all three types of VL device reduced the risk of failed intubation and increased the likelihood of FPS; while channeled and Macintosh-style VL devices also reduced the risk of hypoxemia.

When purchasing airway management equipment for any given hospital/group of affiliated hospitals, airway managers do not select a *group* of devices. Instead, they pick an *individual* device, ideally with the intention of standardizing that single device across all clinical areas where airway management is undertaken﻿ - as is the recommended approach, so that individual and institutional learning may be maximized ⁠ [9]. Choices are likely to be based upon available evidence, device costs, ease of portability, cleaning schedules, training protocols, and previous experience. The majority of non-novice airway managers have experience in DL with a Macintosh-style blade, potentially making a Macintosh-style VL the easiest for developing VL skills.

Two recent studies with sizeable patient numbers have added significant weight to the body of evidence supporting Macintosh-style VL specifically. First, De Jong et al.*’s* study, that included 26,692 intubations in unselected surgical patients [10] reported the benefit of institutional adoption of first-line Macintosh-style VL, demonstrating a significantly increased proportion of patients with ‘easy’ airways when compared with the use of standard DL (98.7% versus 94.3%). Such a clear benefit with regular videolaryngoscopy use is important given the current absence of a perfectly sensitive and specific airway bedside screening test for predicting difficulty [11]. Secondly, the ‘EMMA’ study by Kriege et al., [12] that included 2092 patients requiring tracheal intubation for elective surgery, reported an increase in FPS with Macintosh-style VL compared with DL (94% versus 82%). Despite positive findings such as these, Cook and Aziz stated in their editorial, which endorsed universal videolaryngoscopy, that knowing “which videolaryngoscope or videolaryngoscopes perform best” is a priority question that remains unanswered [13].

We wished to establish whether sufficient evidence can be derived from existing investigations to inform purchasing decisions based upon device-specific performance. To answer this question, we undertook a sub-analysis of the most recent (2022) Cochrane ﻿review by Hansel et al., [7, 8] focusing specifically on Macintosh-style VL devices versus Macintosh DL for elective surgery.

The McGrath MAC and C-MAC are amongst the most common Macintosh-style VLs according to published evidence, and at the time of our re-analysis these were the only devices with sufficient data to support meta-analyses per device. Other Macintosh-style VL devices were represented by up to three studies only [7]. For consistency, we only considered the studies that had been included in the 2022 Cochrane review (all randomized controlled trials) but with the following additional exclusions: (1) studies that did not compare C-MAC VL and Macintosh DL or McGrath MAC VL and Macintosh DL; (2) studies that had been subsequently retracted since their inclusion in the Cochrane review; and, (3) studies that included patients not scheduled for elective surgery.

Separate meta-analyses were performed for McGrath MAC and C-MAC versus Macintosh DL using RevMan 5.4 [14]. Although all outcomes were assessed, there was only a sufficient number of studies (≥ 2) reporting at least one event to enable a meta-analyses for FPS, failed intubation, and esophageal intubation (McGrath MAC only). Most of the studies included for re-analysis were small with less than 100 patients per study arm, with only two studies exceeding that number (Fig. 1). This means that most adverse events, which are generally rare, were not reported in these studies and could therefore not be assessed. Failed intubation and esophageal intubation were assessed using the Peto odds ratio (OR) because this is a superior method for assessing rare events. The risk ratio (RR) was used for FPS.

**Fig. 1:**
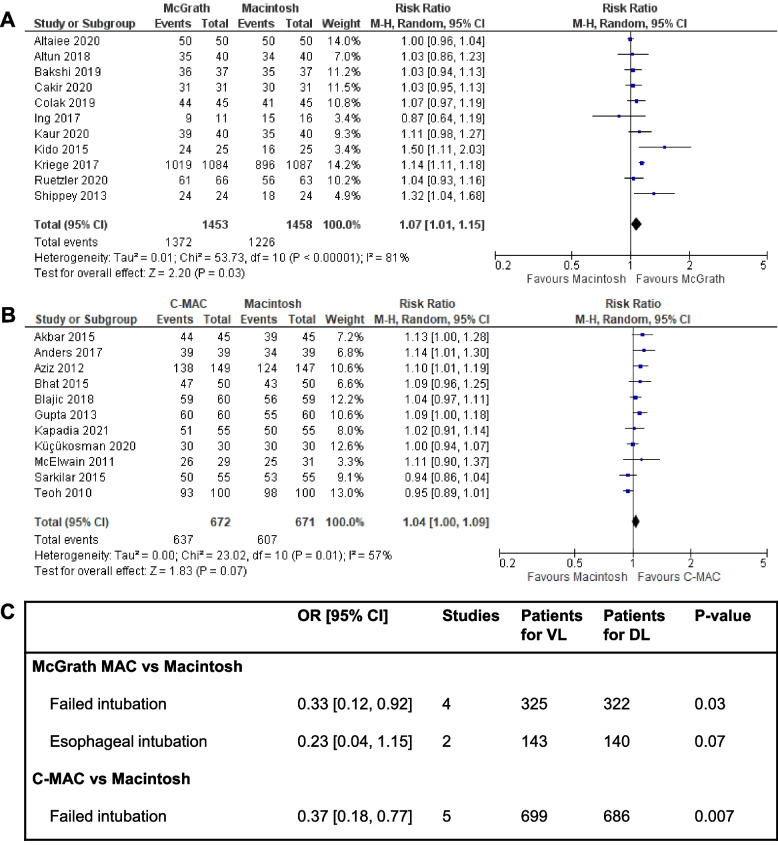
Meta-analyses comparing McGrath MAC VL and C-MAC VL with Macintosh direct laryngoscopy (DL), respectively. Forest plots show FPS given as a risk ratio, also called a relative risk (RR), comparing (**A**) McGrath MAC VL and DL with RR 1.07 [95% CI 1.01, 1.15], *p* = 0.03 and (**B**) C-MAC VL and Macintosh DL with RR 1.04 [1.00, 1.09], *p* = 0.07. The table in (**C**) gives the key statistics of meta-analyses performed for adverse events using the Petos OR for comparison due to these outcomes being rare events. OR < 1.00 favor the VL device because the odds of the adverse event occurring is lower than when using DL. Meta-analysis could not be performed for outcomes reported when there were fewer than two studies reporting at least one event. CI, confidence interval; DL, direct laryngoscopy; FPS, first-pass success; OR, odds ratio; VL, videolaryngoscopy. All studies listed were taken from the 2022 Cochrane review

Both McGrath MAC and C-MAC were found to improve FPS and significantly decrease failed intubations when compared with Macintosh DL (Fig. 1). Although the C-MAC did not quite achieve significance for FPS, results were similar to those reported in the 2022 Cochrane review -﻿ where significance was achieved for the group of Macintosh-style VL devices [7]. In our re-analysis FPS was reported as 84% overall for Macintosh DL and 94% for McGrath MAC (RR 1.07 [95% confidence interval 1.01, 1.15], *p* = 0.03) and it increased from 90% to 95% in the C-MAC studies (RR 1.04 [1.00, 1.09], *p* = 0.07). Reported failed intubations decreased from 3.7% to 1.2% when using McGrath MAC instead of Macintosh DL (OR 0.33 [0.12,0.92], *p* = 0.03) with a similar OR for C-MAC (Fig. 1). Reported esophageal intubations saw a non-significant decrease in events from 3.6% to 0.7% using McGrath MAC instead of Macintosh DL (OR 0.23 [0.04, 1.15], *p* = 0.07). Esophageal intubations could not be assessed for C-MAC because no events were reported in the three small studies included (N ≤ 100 per device).

Care must always be taken with re-analyses of original data. The studies available for this re-analysis were mostly too small (low patient numbers) to assess rare events with a high degree of certainty. Further studies with larger numbers of patients have been conducted since the publication of the 2022 Cochrane review which may help to find the incidence of adverse events when considered collectively. Nevertheless, the presented subgroup analysis re-affirms the 2022 Cochrane review findings in supporting the efficacy of Macintosh-style VL blades and may help inform airway managers’ decisions in choosing which VL device best suits the needs of their institutions.

Whilst this sub-analysis presents the evidence for the efficacy of Macintosh-style VL, the potential benefits to an individual patient may still be limited by lack of device availability/accessibility (though this appears to be improving following the COVID-19 pandemic [15]) and (in)adequacy of staff training in the use of any such device [16]. Regular practice with an institution’s chosen videolaryngoscope is essential in order for the full range of benefits to be conferred to patients.

This subgroup re-analysis does not compare Macintosh-style VL with other blade types such as the hyper-angulated VL. Given the findings of the recent Ruetzler et al. study of 7736 patients undergoing elective surgical procedures that demonstrated superior FPS in intubations conducted with hyper-angulated VL versus DL, hyper-angulated VL merits a greater research focus [17]. This subgroup re-analysis was also intentionally restricted to elective surgical patients, since it represents the most controlled clinical environment where airway management occurs and where VL skills can be most effectively taught to all airway managers [18]. However, it is important to recognize the advantages of VL over DL extend beyond the operating theatre and elective setting, as clearly demonstrated by the recent ‘DEVICE’ and ‘INTUBE’ studies in the emergency department and intensive care environments [19, 20]. Transferability of skills, personnel and the device itself make standardization of VL equipment across an entire hospital/group of hospitals all the more attractive.

This re-analysis has demonstrated largely similar efficacy of the McGrath MAC and C-MAC Macintosh-style VL devices in achieving FPS such that airway managers can select either device based on the evidence provided. We accept that purchasing decisions are likely to remain based on device costs, ease of portability, cleaning schedules, training protocols, and previous experience. However, as VL technology continues to develop, larger comparative studies may be able to more clearly differentiate the *capabilities without complications* of available videolaryngoscopes. In the meantime, this re-analysis provides further information to help airway managers choose a VL device so that the advantages can be delivered to their patients now.

## Data Availability

No datasets were generated or analysed during the current study.
